# A Manual of Diseases of the Nervous System

**Published:** 1900-02

**Authors:** 


A MANUAL OF DISEASES OF THE NERVOUS SYSTEM. By SIR W. R. GOWERS, M. D., F. R. C. P., F. R. S., Consulting Physician to University College Hospital; Physician to the National Hospital for the Paralysed and Epileptic, Queen Square. Third edition, revised and enlarged. Edited by Sir W. R. Gowers and James Taylor, M. A., M. D., F. R. C. P., Senior Assistant Physician to the National Hospital for the Paralysed and Epileptic, Queen Square; Physician to the Northeastern Hospital for Children and to the National Orthopedic Hospital. Vol. I., Diseases of the Nerves and Spinal Cord. With 192 illustrations. Octavo. Philadelphia: P. Blakiston's Sons & Co., 1012 Walnut Street. 1899.

This volume is a thoroughly revised and rewritten work. The newer terms in neurology have been adopted by the author to a slight extent.

The sections on anatomy and physiology of the spinal cord are most complete and painstaking and make a splendid foundation for the chapters on the diseases of the cord. In his description of diseases Gowers has always been most elaborate. Nothing is omitted; everything is given its full value. It is this characteristic, that has made the book such a favorite work of reference. It can never be a text-book, it is too large, too comprehensive. But it is so complete and so encyclopedic that no student of neurology can do without it.

The book is richly illustrated, many of the plates being new. We await with anticipated pleasure the appearance of the second volume, which will present brain and functional disease.

